# Phage cocktails containing a dual-receptor *Phikzvirus* suppress resistance evolution in *Pseudomonas aeruginosa*

**DOI:** 10.1128/aem.02095-25

**Published:** 2026-01-27

**Authors:** Jumpei Fujiki, Kohana Tamamura, Keisuke Nakamura, Tomohiro Nakamura, Yoshiaki Sakata, Nana Kimura, Sayaka Ono, Nozomi Kojima, Keiko Inaba-Hasegawa, Michihito Sasaki, Masaru Usui, Tomohito Iwasaki, Hiroki Ando, Hirofumi Sawa, Hidetomo Iwano

**Affiliations:** 1 Laboratory of Veterinary Biochemistry, Rakuno Gakuen University School of Veterinary Medicine 13022, Ebetsu, Hokkaido, Japan; 2 Laboratory of Phage Biologics, Graduate School of Medicine, Gifu University 12785, Gifu, Japan; 3 Division of Molecular Pathobiology, International Institute for Zoonosis Control, Hokkaido University 12810, Sapporo, Hokkaido, Japan; 4 Institute for Vaccine Research and Development, Hokkaido University 12810, Sapporo, Japan; 5 Laboratory of Food Microbiology and Food Safety, Rakuno Gakuen University School of Veterinary Medicine, Ebetsu, Hokkaido, Japan; 6 Department of Food Science and Human Wellness, College of Agriculture, Food and Environment Science, Rakuno Gakuen University 13022, Ebetsu, Hokkaido, Japan; 7 Center for One Medicine Innovative Translational Research (COMIT), Institute for Advanced Study, Gifu University 12785, Gifu, Japan; 8 Venture Unit Engineered Phage Therapy, Discovery Accelerator, Astellas Pharma Inc., Tsukuba, Ibaraki, Japan; 9 Arrowsmith Inc., SakuLab-Tsukuba, Tsukuba, Ibaraki, Japan; 10 International Collaboration Unit, International Institute for Zoonosis Control, Hokkaido University 12810, Sapporo, Hokkaido, Japan; 11 Global Virus Network, Baltimore, Maryland, USA; 12 One Health Research Center, Hokkaido University 12810, Sapporo, Hokkaido, Japan; Universidad de los Andes, Bogotá, Colombia

**Keywords:** phage therapy, phage cocktail, phage resistance, antimicrobial resistance (AMR), fitness, trade-offs

## Abstract

While phage therapy is one of the promising strategies against antimicrobial resistant infections by *Pseudomonas aeruginosa*, the rapid emergence of phage-resistant variants remains a significant barrier to its long-term clinical efficacy, reflecting the constant evolutionary arms race between phages and their hosts. Here, we first characterized ΦBrmt, a *Phikzvirus* phage previously isolated from an LPS-defective *P. aeruginosa* mutant of the Pa12 strain. Whole-genome sequencing of ΦBrmt-resistant variants derived from the Pa12 strain (Pa12 mt^ΦBrmt^) revealed mutations in genes for type IV pili and flagellar biosynthesis, resulting in decreased motility. To identify its receptors, we tested ΦBrmt against a panel of knock-out mutants, revealing that it failed to infect a *ΔpilA*/*ΔfliC* double mutant, despite being able to infect each single mutant. Transmission electron microscopy revealed that ΦBrmt adsorbed to the flagella of the Pa12 WT, whereas this adsorption was abolished on the phage-resistant mutants Pa12 mt^ΦBrmt^. In contrast, *Pbunavirus* ΦS12-3 and ΦR26 were unable to infect the Δ*galU* mutant but formed clear plaques on the Δ*pilA* and Δ*fliC* strains. A cocktail combining the pili/flagella-targeting ΦBrmt with an LPS-targeting *Pbunavirus* phage significantly suppressed the emergence of phage-resistant variants *in vitro* against representative clinical isolates when compared to single-phage treatments. Our findings demonstrate that combining phages targeting distinct classes of bacterial receptors is a powerful strategy to limit resistance development, indicating that identifying the receptor genes utilized by *Pseudomonas* phages can be the rational starting point for such design.

**IMPORTANCE:**

Phage resistance limits the clinical efficacy of phage therapy against *P*. aeruginosa, a major antimicrobial-resistant pathogen. To address this, we demonstrate that a cocktail combining phages targeting distinct class of receptors effectively suppresses resistance. Through genetic analysis of resistant mutants, we first identified that the phage Brmt (ΦBrmt) uses both Type IV pili and flagella as receptors; a double mutant deficient in both *pilA* and *fliC* became completely resistant to infection. We then combined ΦBrmt with an LPS-targeting *Pbunavirus* phage, whose receptor was confirmed using a *ΔgalU* mutant. This receptor-diverse cocktail significantly suppressed the emergence of resistant variants across 10 diverse clinical isolates *in vitro* compared to single-phage treatments. These results underscore the importance of receptor-based molecular characterization as a critical first step in rational phage cocktail design. Our findings provide mechanistic insights into phage-host interactions and highlight a practical strategy for constructing receptor-diverse phage combinations to delay resistance evolution and enhance therapeutic robustness.

## INTRODUCTION

Antimicrobial resistance (AMR) in pathogenic bacteria presents a considerable threat to public health, with projections estimating 10 million annual deaths by 2050 without effective global action (1). *Pseudomonas aeruginosa,* one of the critical ESKAPE pathogens (*Enterococcus faecium, Staphylococcus aureus, Klebsiella pneumoniae, Acinetobacter baumannii, Pseudomonas aeruginosa*, and *Enterobacter* spp.), is a priority for new antibiotic development and poses a significant challenge in both human and veterinary medicine due to its frequent multi-drug resistance (2, 3). This has led to renewed interest in phage therapy as a promising alternative. Bacteriophages are viruses that specifically infect bacteria, utilizing a lytic lifecycle to replicate and ultimately kill their host (4, 5). While phage therapy has a long history of use in Eastern European countries (4, 5) and recent successful clinical trials in the Western countries have highlighted its potential against AMR infections (6–8), bacterial phage resistance has emerged during actual phage therapy cases (9, 10). Therefore, phage therapy faces a significant barrier that threatens its long-term clinical efficacy. To address this, phage cocktails—combinations of multiple phages—are generally used to suppress the emergence of resistance (4, 9, 10); therefore, establishing a rational design framework for these cocktails against each bacterial species is essential.

Pbunaviruses are promising lipopolysaccharide (LPS)-targeting phages for therapy because *Pbunavirus* ΦBrSP1 and ΦS12-3 exhibit efficient lytic activities and have broad host ranges against domestic and companion animal-related *P. aeruginosa* strains (11–13). *Pbunavirus* phages adsorb onto host cells by utilizing the *O*-antigen in the LPS structure (14). But once mutations occur in the *wzy* gene that encodes the LPS repeat unit polymerase, *P. aeruginosa* can escape *Pbunavirus* phage infections (14). In addition, *galU*, which is essential for the complete core oligosaccharide biosynthesis, plays a critical role in phage resistance acquisition (15). Shen et al. demonstrated that deletion of *galU* from the *P. aeruginosa* chromosome by MutL, which is associated with the DNA mismatch repair system, during phage infection leads to phage resistance, as *galU* deletion induces a lack of *O*-antigen polysaccharide and the absence of a phage receptor (16). In fact, *Pbunavirus* ΦS12-3 never infects *galU*-deficient strains of the *P. aeruginosa* veterinary isolate Pa12 (17, 18). As *hmgA*, which converts red-colored homogentisic acid to 4-maleylacetoacetate, is located very close to *galU* on the chromosome, phage-resistant variants of *P. aeruginosa* with large chromosomal deletions exhibit a brown colored phenotype, called the brown mutant (Brmt) (16).

In Gram-negative bacteria, while the LPS layer serves as a major receptor for many phages, a diverse array of other cell surface structures, including outer membrane proteins, pili, and flagella, are also known to function as attachment sites. For instance, jumbo phages, exemplified by members of the *Phikzvirus*, are reported to utilize type IV pili as primary receptors, and deletion or mutation of a key pilus biosynthesis gene (e.g., *pilA, pilB, pilT,* and *pilZ*) completely abolishes their infectivity (19–21). Similarly, the single polar flagellum has been suggested as a receptor for certain *Pseudomonas* phages (22, 23). The most well-characterized example is the *Pseudomonas* virus ΦCTX, which is classified as a flagellotropic phage because its infection is blocked by antisera against flagellin or specific flagellin-derived peptides (24). However, while the molecular mechanisms of flagellotropic phage infection are relatively well-studied in *Escherichia coli*, with some studies identifying the necessity of counter-clockwise flagellar rotation and even secondary receptors (23), such detailed understanding in *Pseudomonas* is lacking. With the notable exception of ΦCTX, there is no definitive evidence of flagella serving as the primary receptor for other *Pseudomonas* phages. Elucidating these infection pathways is, therefore, critical, not only for a deeper understanding of phage diversity but also for enabling the rational design of effective phage cocktails.

The accumulation of mutations in phage receptor-encoding genes is a primary mechanism of phage resistance, often altering bacterial phenotypes due to structural and functional changes in the receptor molecules (9, 10, 14, 15). Although cocktails combining diverse *Pseudomonas* phages can delay the emergence of such resistance *in vitro* and have been applied in clinical settings (9, 12, 13, 25), there remains a notable lack of reports verifying the efficacy of phage cocktails rationally assembled based on the detailed molecular identification of their specific host receptors. Therefore, in the present study, we first aim to determine the molecular infection mechanisms for receptors of *Pbunavirus* and *Phikzvirus* phages we previously isolated (12, 13, 26, 27). Based on this understanding, we then seek to validate a rationally designed phage cocktail.

## MATERIALS AND METHODS

### *Pseudomonas aeruginosa* strains and bacteriophages

*P. aeruginosa* veterinary isolates, which were previously isolated from dog skin infection sites (assigned as Pa) (13, 26), were used in the present study. In addition, the *P. aeruginosa* PAO1 strain derived from American Type Culture Collection (ATCC, Manassas, VA) was used to generate a panel of knock-out mutants (Table S1). *Pseudomonas* viruses ΦS12-3, ΦR12, and ΦR26 used in the present study were also previously isolated from sewage water collected from a sewage treatment plant in the city of Ebetsu, Hokkaido, Japan. These phages were categorized as *Pbunavirus* PB1-like phages (12, 13, 26). The *Pseudomonas* virus ΦBrmt, classified as a *Phikzvirus* phage, was previously isolated from wastewater samples collected in Hokkaido and Kochi prefectures using the Pa12 Brmt strain (27). Pharokka (28) was used to annotate the ΦBrmt genome.

### Bacteriophage preparation and plaque assay

For downstream assays, phages were propagated by the plate lysate method as described elsewhere (29). In brief, an aliquot of the propagating strain, *P. aeruginosa* Pa12, grown in LB medium (Becton Dickinson) was combined with an aliquot of phages (ΦS12-3, ΦR12, ΦR26, and ΦBrmt) and added to 3 mL of LB top agar containing 0.5% or 0.25% agarose ME. The mixture then was overlaid on an LB agar plate. After overnight incubation of the plate at 37°C, 3 mL of SM buffer (10 mM MgSO_4_, 100 mM NaCl, 0.01% gelatin, and 50 mM Tris-HCl pH 7.5) was added to the plate, and the plate was incubated at room temperature for 1 to 2 h with shaking. The overlaid top agar was scraped off and homogenized with SM buffer using a colony spreader. The collected homogenate was centrifuged at 6,500 *g* for 15 min at 4°C to remove remaining bacteria and debris. Resultant supernatants were passed through 0.45-µm membrane filter (ADVANTEC, Tokyo, Japan) and purified using an Amicon Ultra-membrane filter (Merck, Darmstadt, Germany) based on the phage on tap (PoT) method described by Bonilla et al. (30). The phage titer was calculated as the number of plaques in a plaque assay using Pa12, in accordance with previous reports (29), and is represented as plaque- forming units per milliliter (PFU/mL).

### Efficiency of plating assay

Phages’ host range and infectivity against target strains were determined by the EoP method in accordance with previous reports (29). In brief, target strains of *P. aeruginosa* were grown in LB medium overnight at 37°C with shaking 3 mL of LB top agar containing 100 µL of *P. aeruginosa* strains was overlaid on an LB agar plate. Thereafter, 3 µL of diluted phage aliquots (10^7^ to 10^1^ PFU/mL) in SM buffer was dropped onto overlaid LB agar plates to observe the lytic activity of phages by plaque formation. EoP values represent PFU using the specific *P. aeruginosa* strain/PFU using the propagating strain Pa12.

### Isolation of phage-resistant variants of *P. aeruginosa*

Phage-resistant Pa12 variants against ΦBrmt (Pa12 mt^ΦBrmt^) were selected by liquid culture. ΦBrmt (10^9^ PFU/mL) was inoculated into a Pa12 overnight culture in 4 mL of fresh LB medium at a multiplicity of infection (MOI) of 0.1. After overnight incubation with shaking at 37°C, 20 µL of the culture was passaged to 4 mL of fresh LB medium with 40 µL of ΦBrmt every 1 or 2 days. After 1 week of co-culture with ΦBrmt and Pa12, the culture was collected and streaked onto an LB agar plate. After overnight incubation at 37°C, a single colony was picked up. This cloning step was repeated three times to ensure that they were clonal isolates. Thereafter, picked colonies were grown in LB medium to amplify and store the variants. Phage sensitivity of the isolated clones was determined by EoP assays as described above, and the lytic activity of phages was evaluated by plaque formation.

### Adsorption assay

Adsorption rates of ΦBrmt toward the propagating strain Pa12 and Pa12mt^ΦBrmt^ were determined as described in a previous report (31). In brief, ΦBrmt in SM buffer (10^6^ PFU/mL) was suspended 1:1 with *P. aeruginosa* target strains at a MOI of 0.01. After a 10-min incubation at room temperature, the samples were immediately centrifuged at 10,000 *g* for 1 min, and the resultant supernatant containing unabsorbed phages was used for the plaque assay described above.

### Whole-genome sequencing

DNA extracted from Pa12mt^ΦBrmt^ was submitted to Bioengineering Lab. Co., Ltd., for whole-genome sequencing. The samples were sequenced as 200 base paired-end reads on a DNBSEQ-G400 platform (MGI tech, Shinsen, China). The obtained reads were used for comparative genome analysis with a previously determined Pa12 parental chromosome sequence (AP024513) using BV-BRC (https://www.bv-brc.org/) to identify single-nucleotide variants.

### Electron microscopic analysis

Electron microscopic imaging was performed as described previously (13, 31). Purified ΦBrmt samples were loaded onto copper grids (EMJapan, Tokyo, Japan). The grids were washed with SM buffer twice and stained with 2% uranyl acetate. In order to observe the flagella structure and attachment of phages to *P. aeruginosa*, an electron micrograph was acquired in accordance with a previous report (32). *P. aeruginosa* strains were adsorbed onto collodion-carbon-coated 400-mesh copper grids (10 min) with or without ΦBrmt and fixed on a drop of 1% glutaraldehyde in 30 mM HEPES buffer, pH 7.3, for 5 min. The grid was then immediately stained with one drop of a 2% aqueous uranyl acetate solution for 60 s. Stained samples were observed with a Hitachi HT7700 transmission electron microscope (Hitachi Ltd., Tokyo, Japan) at 80 kV.

### Swimming and twitching activity assay

Swimming and twitching activity were assessed in accordance with previous reports (33). In brief, 2 µL of standardized *P. aeruginosa* strains (OD_590_ = 0.6) was spotted onto the center of 0.25% LB agar plates for swimming activity assays. After overnight incubation at 37°C, swimming zone diameters (from center to the outermost part of the swimming zone) were evaluated. For the twitching activity assay, *P. aeruginosa* strains were inoculated on the bottom of 1% LB agar by stabbing with a P10 pipette tip. Thereafter, plates were incubated at 37°C overnight and stained with 1% crystal violet solution (Nacalai Tesque, Kyoto, Japan) for 30 min. Excess dye was washed away with water, and twitching zone diameters were quantified.

### Construction of knockout strains

Gene deletion mutants were generated by homologous recombination between the target gene and an antibiotic resistance cassette, essentially following the method of Lesic et al. (34), using *P. aeruginosa* PAO1 carrying pUCP18-RedS (custom-synthesized by Invitrogen, Thermo Fisher Scientific, Waltham, MA). Kanamycin resistance (Km^R^) and gentamicin resistance (Gm^R^) cassettes were PCR-amplified from pMW218 (Nippon gene, Tokyo, Japan) and pJN105 (custom-synthesized by Invitrogen), respectively. Recombination fragments were generated by one-step PCR using 100-nt primers and either Km^R^ or Gm^R^ cassette as a template. Each primer consisted of 85-nt of homology to the upstream (5′ primer) or downstream (3′ primer) flanking region of the target gene at the 5′ end, and 15-nt homology to the corresponding end of the resistance cassette at the 3′ end. For *galU* deletion, a two-step PCR was used. First, three fragments were independently amplified: the Km^R^ cassette, and ~200 bp upstream and downstream flanking regions of *galU* from PAO1 genomic DNA. These fragments were then fused by overlap extension PCR using outer primers targeting the 5′ end of the upstream fragment and the 3′ end of the downstream fragment. The final PCR product was purified (QIAquick PCR Purification Kit, QIAGEN, Hilden, Germany) and concentrated by ethanol precipitation. Primers used are listed in Table S2.

PAO1/pUCP18-RedS was cultured in LB with carbenicillin at 37°C to an OD_600_ of 0.4, induced with 0.2% L-arabinose, and incubated for 1.5 h. Cells were harvested by centrifugation at 8,000 × *g* for 5 min and washed twice with SMEB buffer (1 mM HEPES [pH 7.0], 1 mM MgCl₂, 300 mM sucrose) at room temperature. The pellet was resuspended in SMEB buffer at 1/120 of the original culture volume and used as competent cells. Electroporation was performed with 100 µL of competent cells and 2–5 µg of PCR product under the following conditions: 2.5 kV, 25 µF, and 200 Ω. Cells were recovered in LB at 37°C for 1 h and plated on LB agar with kanamycin (300 µg/mL) or gentamicin (75 µg/mL). Mutants were streaked on LB agar with 10% sucrose and incubated overnight to cure the pUCP18-RedS. Plasmid loss was verified by streaking on LB agar with carbenicillin.

### Monitoring the growth of Pa12 with or without phage cocktails

The lytic activity of phage cocktails composed of *Pbunavirus* ΦS12-3, ΦR12, ΦR26, and *Phikzvirus* ΦBrmt against *P. aeruginosa* veterinary isolates was evaluated in turbidity assays by monitoring the OD_590_ for 24 h and 48 h using a plate reader (Sunrise Rainbow Thermos RC; TECAN, Austria) as previously reported (17, 31). In brief, phage cocktails or each component phage were inoculated into exponentially growing *P. aeruginosa* cultures in a 96-well plate at a MOI of 0.01 (In the cocktail experiments, a combined MOI of 0.01 was used, with equal proportions of each phage). The density of the culture was monitored using a plate reader every 1 h. After 48 h of inoculation (hpi), the number of viable *P. aeruginosa* cells in the culture was determined by plating on LB agar and expressed as colony-forming units per milliliter (CFU/mL).

### Statistical analysis

Statistical analysis was performed using Dunnett’s test based on one-way ANOVA to compare between three or more groups from at least three experiments. Values of *P* less than 0.05 were considered statistically significant. Where applicable, statistical significance is indicated by asterisks, with *P < 0.05, **P < 0.01, ***P < 0.001, and ****P < 0.0001. All statistical analyses were performed using GraphPad Prism Version 10.4.2 (534).

## RESULTS

### Morphology of *Pseudomonas* virus against the Brmt strain

**Fig 1:**
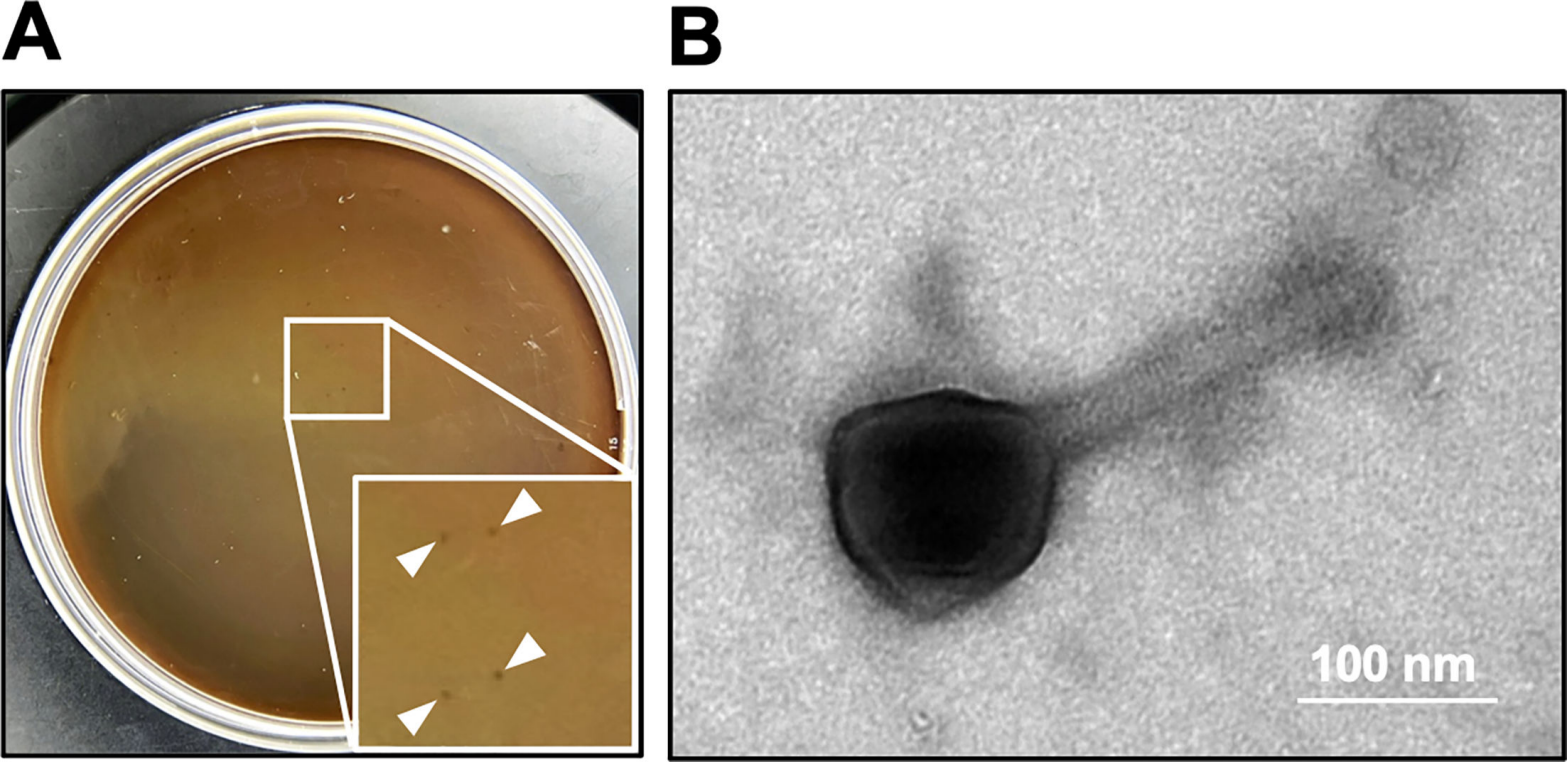
Plaque formation on Brmt of a *Pseudomonas* virus, ΦBrmt, and its morphology. (**A**) Plaques representing *Pseudomonas* virus ΦBrmt are observed on a lawn of the Pa12 Brmt strain. (**B**) Electron microscopic image showing phage particles exhibiting *Myoviridae* characteristics. Bar = 100 nm.

Previously, we tried to isolate a novel virulent phage by using Brmt as the host strain in order to increase the chances of finding non-LPS targeting phages. The isolated phage, designated ΦBrmt, was classified as a *Phikzvirus* phage based on phylogenetic analysis of its whole-genome sequence (27). ΦBrmt exhibited plaque-forming activity against the Pa12 Brmt strain (Fig. 1A), suggesting that its infection is independent of the O-antigen structure of the target *P. aeruginosa* strains. The purified ΦBrmt was subjected to electron microscopic imaging, which identified phage particles revealing a contractile tail and a relatively long polyhedral head (Fig. 1B), characteristic of the Myoviridae morphotype according to previous Ackermann’s classification (35). In addition, annotation analysis showed that ΦBrmt has no known temperate phage-related genes such as integrase in the genome, suggesting that ΦBrmt is a virulent phage (Table S3). Furthermore, EoP assays indicated that infectivity of ΦBrmt did not differ markedly between the Pa12 parental strain and the Pa12 Brmt strain (Fig. S1).

### Isolation of phage resistant variants of Pa12 against ΦBrmt

**Fig 2:**
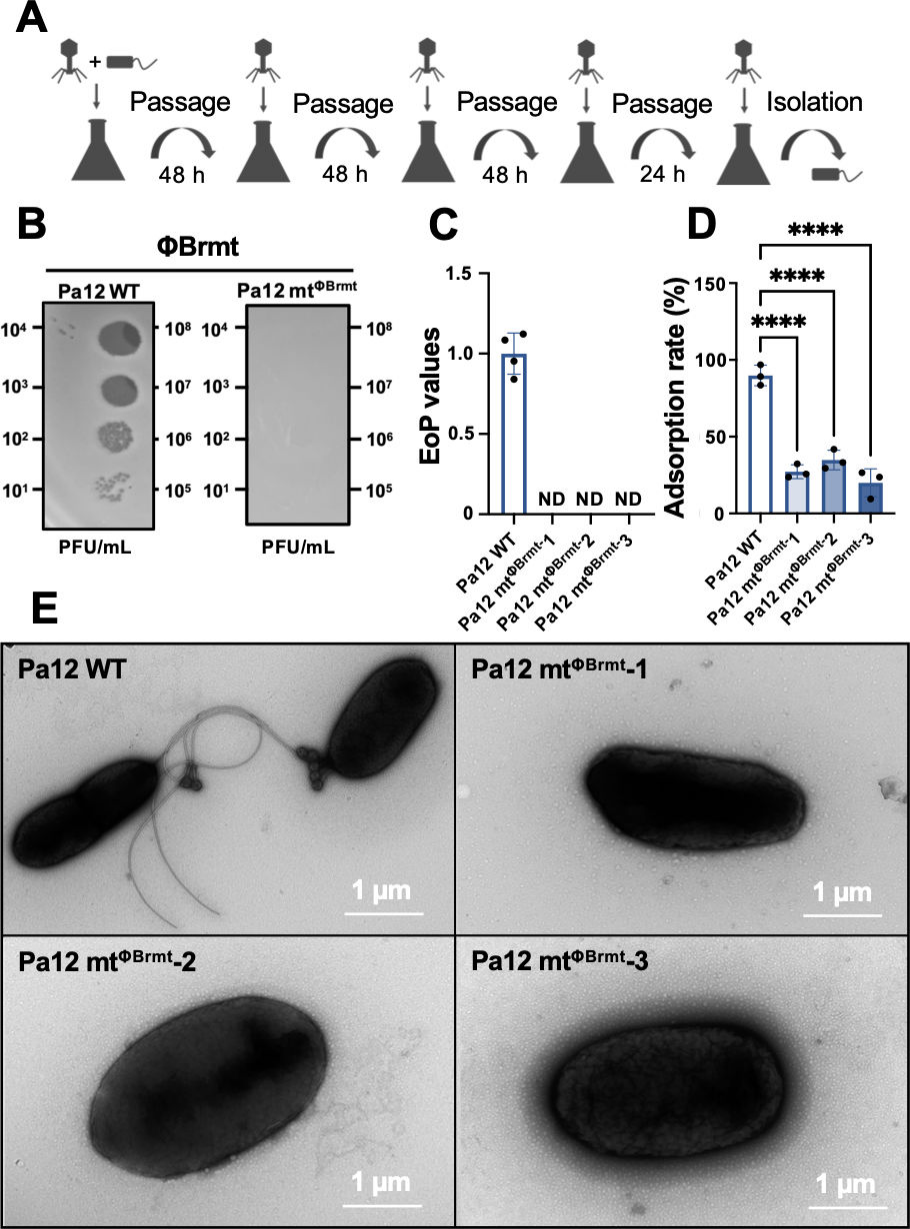
Isolation of Pa12-resistant variants against ΦBrmt and determination of phage sensitivity and adsorption. (**A**) Schematic representation of liquid-based isolation of Pa12 variants exhibiting ΦBrmt resistance. (**B**) Representative images of EoP assays using ΦBrmt against Pa12 mt^ΦBrmt^ −1. (**C**) EoP values of ΦBrmt infectivity against Pa12 mt^ΦBrmt^ −1, 2, and 3, indicated as fold changes compared to WT. ND, plaques not detected. (**D**) Adsorption rate of ΦBrmt on Pa12 WT and Pa12 mt^ΦBrmt^ −1, 2, and 3, presented as means ± SD (*n* = 3). Significance against WT was analyzed by Dunnett’s test based on one-way ANOVA: ****P < 0.0001. (**E**) Electron microscopic images showing ΦBrmt-resistant *P. aeruginosa* and WT, and adsorption of ΦBrmt against WT strain. Bar = 1 µm.

To assess the ΦBrmt mechanisms of *P. aeruginosa* infection, we selected Pa12 phage-resistant variants after co-culture of Pa12 and ΦBrmt as shown in Fig. 2A. After co-culture, we streaked and cloned three variants for downstream assays. In the EoP assay, these three variants diminished phage susceptibility against ΦBrmt (Fig. 2B and C), so we designated these variants as Pa12 phage-resistant mutants against ΦBrmt (Pa12 mt^ΦBrmt^) −1, 2, and 3. In addition, adsorption assays clearly showed that Pa12 mt^ΦBrmt^ decreased the adsorption rate of ΦBrmt significantly, suggesting that Pa12 mt^ΦBrmt^ harbors structural changes in receptor molecules for phage adsorption (Fig. 2D). Subsequent observations using electron microscopy revealed that ΦBrmt particles were adsorbed onto the flagellar structures of Pa12 WT as shown in Fig. 2E. In contrast, the three Pa12 mt^ΦBrmt^ strains lacked visible flagella, and no phage particles were observed attached to flagella after mixing with ΦBrmt.

### Whole genome sequencing of Pa12 mt^ΦBrmt^

**Fig 3:**
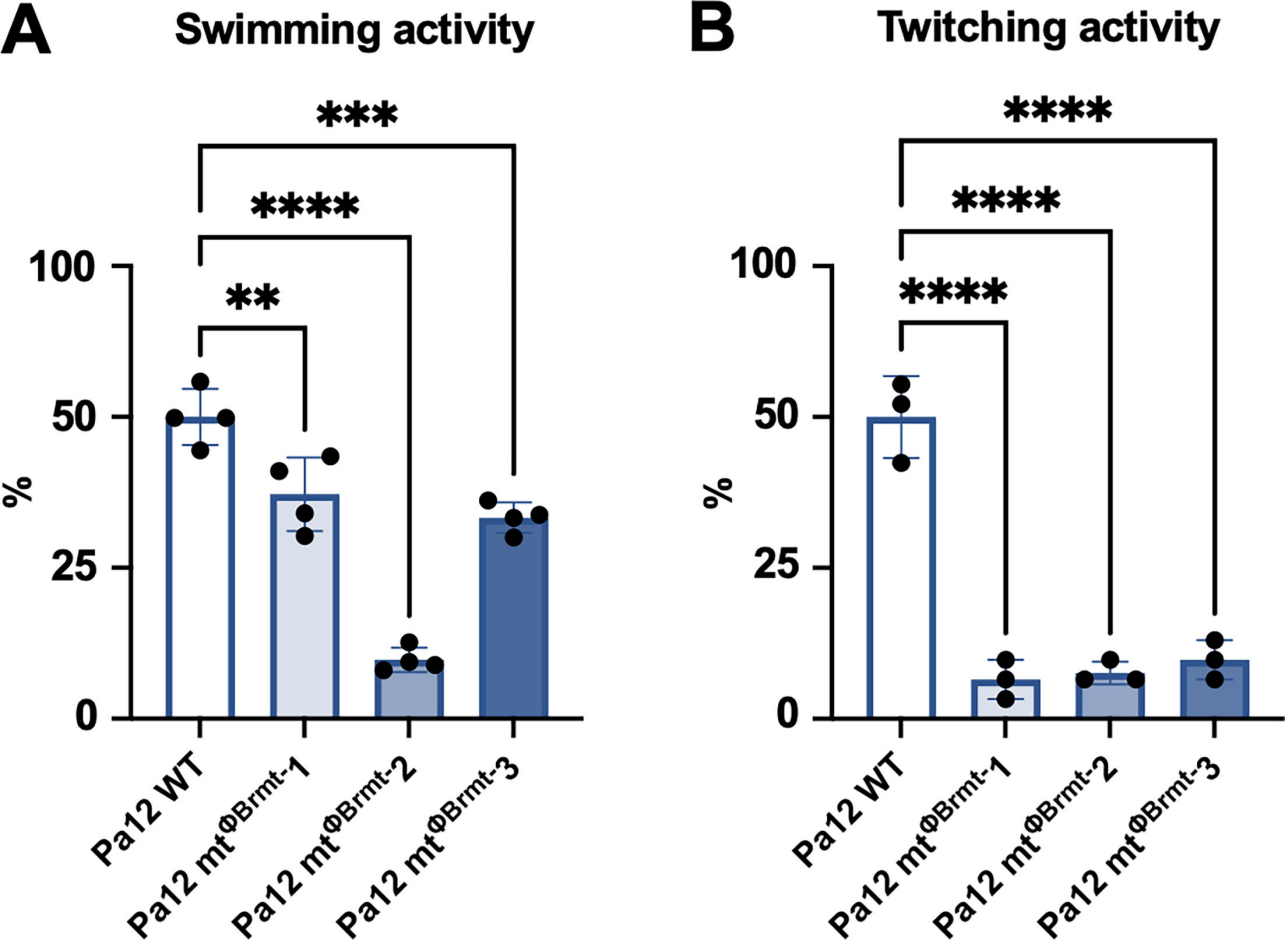
Swimming and twitching motility of Pa12 mt^ΦBrmt^. (**A**) Swimming motility of WT and Pa12 mt^ΦBrmt^ −1, 2, and 3 indicated as fold changes compared to WT and presented as means ± SD (*n* = 4). Significance against WT was analyzed by Dunnett’s test based on one-way ANOVA: **P < 0.01, ***P < 0.001, ****P < 0.0001. (**B**) Twitching motility of WT and Pa12 mt^ΦBrmt^ −1, 2, and 3 indicated as fold changes compared to WT and presented as means ± SD (*n* = 3). Significance against WT was analyzed by Dunnett’s test based on one-way ANOVA: ****P < 0.0001.

The three Pa12 mt^ΦBrmt^ strains were subjected to whole-genome sequencing to identify the genetic mutations. As shown in Table 1, all Pa12 mt^ΦBrmt^ had genetic mutations in flagellar biosynthesis genes such as *fliP*, *flhA*, and *fliF* for Pa12 mt^ΦBrmt^ 1, 2, and 3, respectively, which is consistent with the absence of flagellar structures observed in electron microscopy, in contrast to Pa12 WT. In addition, Pa12 mt^ΦBrmt^ had genetic mutations in type IV pilus biosynthesis genes such as *pilY1*, *pilJ*, and *pilA* for Pa12 mt^ΦBrmt^ 1, 2, and 3, respectively. As expected, no mutations were detected in LPS biosynthesis-related genes in Pa12 mt^ΦBrmt^. Furthermore, swimming and twitching activity assays clearly showed significant decreases in motility in Pa12 mt^ΦBrmt^ 1, 2, and 3 compared with parental Pa12 WT (Fig. 3A and B).

**TABLE 1:** Genetic mutations^*a*^ in Pa12 mt^ΦBrmt^

Variant	Position	Type^*b*^	Reference	Alteration	Frequency	Effect	Gene	Product
Pa12 mt^ΦBrmt^-1	4145810	Deletion	GA	G	99.6%	Frameshift	*fliP*	Flagellar biosynthetic protein FliP
	5222525	Complex	TCGA	GAAG	100%	Stop	*pilY1*	Type IV pilus biogenesis factor PilY1
Pa12 mt^ΦBrmt^-2	451020	Deletion	TGACCGTGGCCGC	T	100%	Inframe deletion	*pilJ*	Type IV pilus biogenesis factor PilJ
	4139331	Deletion	AC	A	100%	Frameshift	*flhA*	Flagellar biosynthesis protein FlhA
	5350446	SNV	C	G	99.5%	Missense	*moeB*	Molybdopterin-synthase adenylyltransferase MoeB
Pa12 mt^ΦBrmt^-3	4443468	Deletion	ACGCTGT	A	96.7%	Inframe deletion	*fliF*	Flagellar M-ring protein FliF
	5188218	SNV	G	A	100%	Stop	*pilA*	Type IV pilus major pilin protein PilA

^*a*^ Genetic mutations detected in Pa12 mt^ΦBrmt^-1, 2, and 3 located in the coding sequences.

^*b*^ Complex, multiple nucleotide variants occurring at a single position or adjacent positions. SNV, single nucleotide variant.

### Host spectrum of ΦBrmt

To evaluate the ΦBrmt host spectrum, we performed EoP assays against 39 strains of veterinary *P. aeruginosa* clinical isolates. Table 2 shows that ΦBrmt harbored plaque forming activity against 11/39 (28.2%) of *P. aeruginosa* strains, which was a relatively narrow host spectrum compared with *Pbunavirus* ΦS12-3 (30.8%) and ΦR26 (46.2%), but wider than that of ΦR12 (20.5%), which is consistent with a previous report (13).

**TABLE 2:** Host ranges of ΦBrmt and *Pbunavirus* phages based on EoP^*a*^

Strain	ΦBrmt	ΦS12-3	ΦR12	ΦR26	Strain	ΦBrmt	ΦS12-3	ΦR12	ΦR26
Pa01	+	+	−	+	Pa49	−	−	−	+
Pa04	+	−	−	−	Pa50	−	−	−	+
Pa07	−	−	−	−	Pa51	−	−	−	−
Pa08	−	−	−	−	Pa52	+	+	−	+
Pa11	−	+	+	−	Pa53	−	−	−	+
Pa12	+	+	+	+	Pa54	−	−	−	−
Pa14	+	+	+	+	Pa56	+	+	−	+
Pa16	−	−	−	−	Pa57	−	+	+	+
Pa17	−	−	−	−	Pa58	−	−	−	−
Pa18	−	−	−	−	Pa59	−	+	+	−
Pa22	−	−	−	−	Pa60	+	−	−	+
Pa25	+	−	−	−	Pa61	+	+	+	+
Pa26	−	−	−	+	Pa63	−	−	−	+
Pa27	−	−	−	−	Pa64	−	+	+	+
Pa29	−	−	−	−	Pa65	−	−	−	+
Pa34	−	−	−	−	Pa66	−	−	−	−
Pa38	−	−	−	−	Pa67	−	+	+	−
Pa42	+	+	−	+	Pa68	−	−	−	−
Pa43	−	−	−	+	Pa70	+	−	−	+
Pa44	−	−	−	−	Range	28.2%	30.8%	20.5%	46.2%

^*a*^ Plaque forming activities of ΦBrmt, ΦS12-3, ΦR12, and ΦR26 against *P. aeruginosa* clinical isolates. +, a plaque was detected; −, a plaque was not detected.

### Receptor determination of *Pbunavirus* ΦR26, ΦS12-3, and *Phikzvirus* ΦBrmt

**Fig 4:**
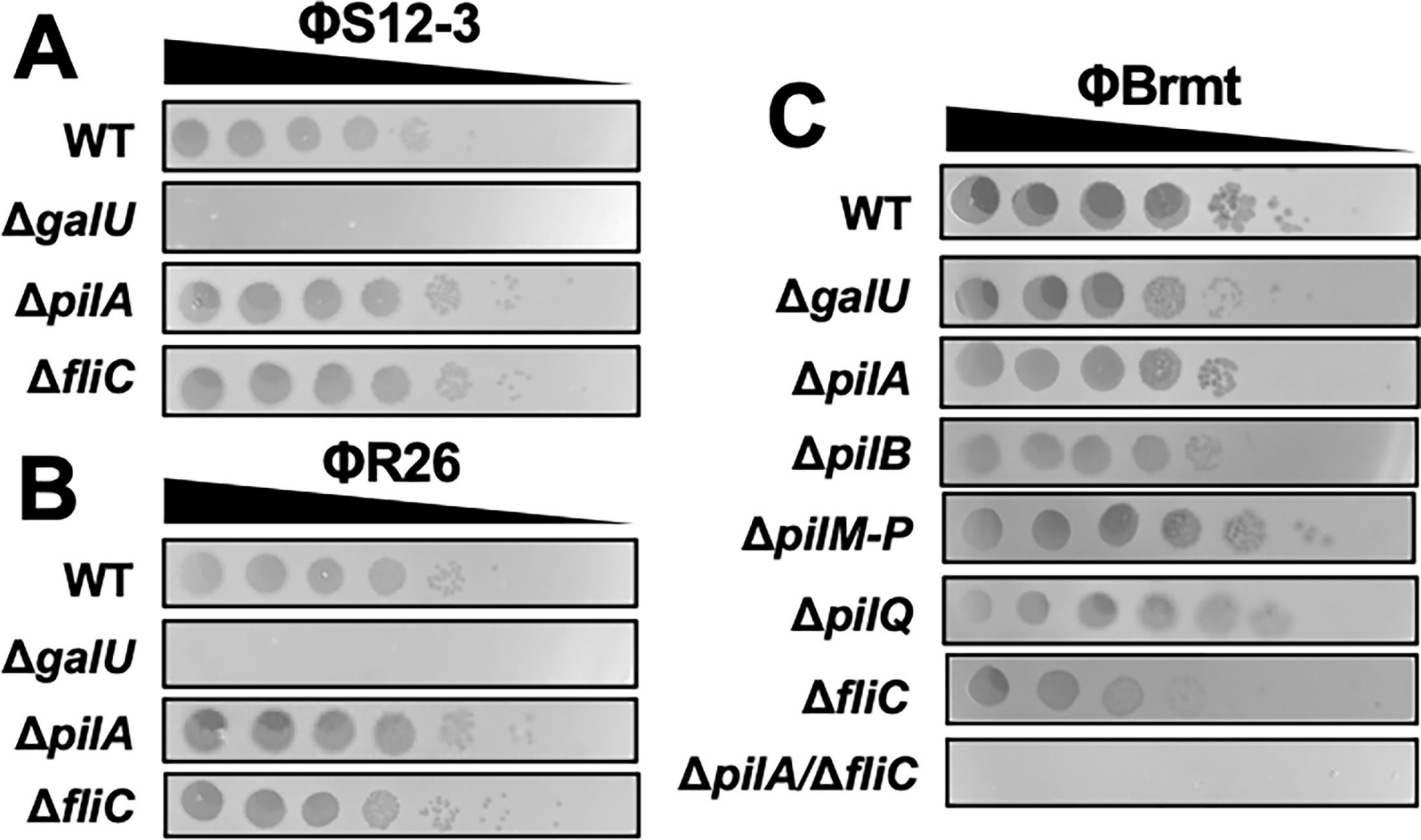
Infectivity of ΦS12-3, ΦR26, and ΦBrmt against knock out strains of *P. aeruginosa* PAO1. Infectivity of *Pbunavirus* phages (ΦS12-3 and ΦR26) against LPS-defective (Δ*galU*), pili-defective (Δ*pilA*), and flagella-defective (Δ*fliC*) strains (**A and B**). Infectivity of ΦBrmt against LPS-defective (Δ*galU*), pili-defective (Δ*pilA*, Δ*pilB*, Δ*pilM-P*, and Δ*pilQ*), and flagella-defective (Δ*fliC*) strains (**C**). The Δ*pilA*/Δ*fliC* strain is a double-knockout mutant of PAO1.

To determine the host receptors for the *Pbunavirus* ΦR26 and ΦS12-3, as well as the *Phikzvirus* ΦBrmt, we evaluated the plaque-forming ability of these phages using a panel of *P. aeruginosa* PAO1 knockout mutants. ΦR12 was excluded from this analysis because it does not infect the PAO1 strain. Consistent with previous reports, both ΦR26 and ΦS12-3 lost infectivity against the PAO1 *ΔgalU* mutant, indicating that they are LPS-targeting phages (Fig. 4A and B). In contrast, their infectivity was retained in the *ΔpilA* and *ΔfliC* mutants, suggesting that these structural components are not required for infection by ΦR26 or ΦS12-3. For ΦBrmt, no substantial reduction in plaque formation was observed with single deletions of *galU*, or with genes involved in pilus (*pilA, pilB, pilQ, pilM-P*) or flagellar (*fliC*) biosynthesis (Fig. 4C). However, the double-knockout mutant lacking both *pilA* and *fliC* completely lost susceptibility to ΦBrmt. These results suggest that ΦBrmt requires either pili or flagella as a receptor for successful infection. This finding is also supported by the mutation analysis of the three Pa12 mt^ΦBrmt^ strains, all of which harbored mutations in genes related to both pili and flagella biosynthesis (Table 1). In addition, these results strongly suggest that *Phikzvirus* phages such as ΦBrmt and *Pbunavirus* phages such as ΦS12-3 and ΦR26 utilize distinct receptor classes for infection on *P. aeruginosa*.

### Infectivity of *Pbunavirus* phages toward Pa12 mt^ΦBrmt^

**Fig 5:**
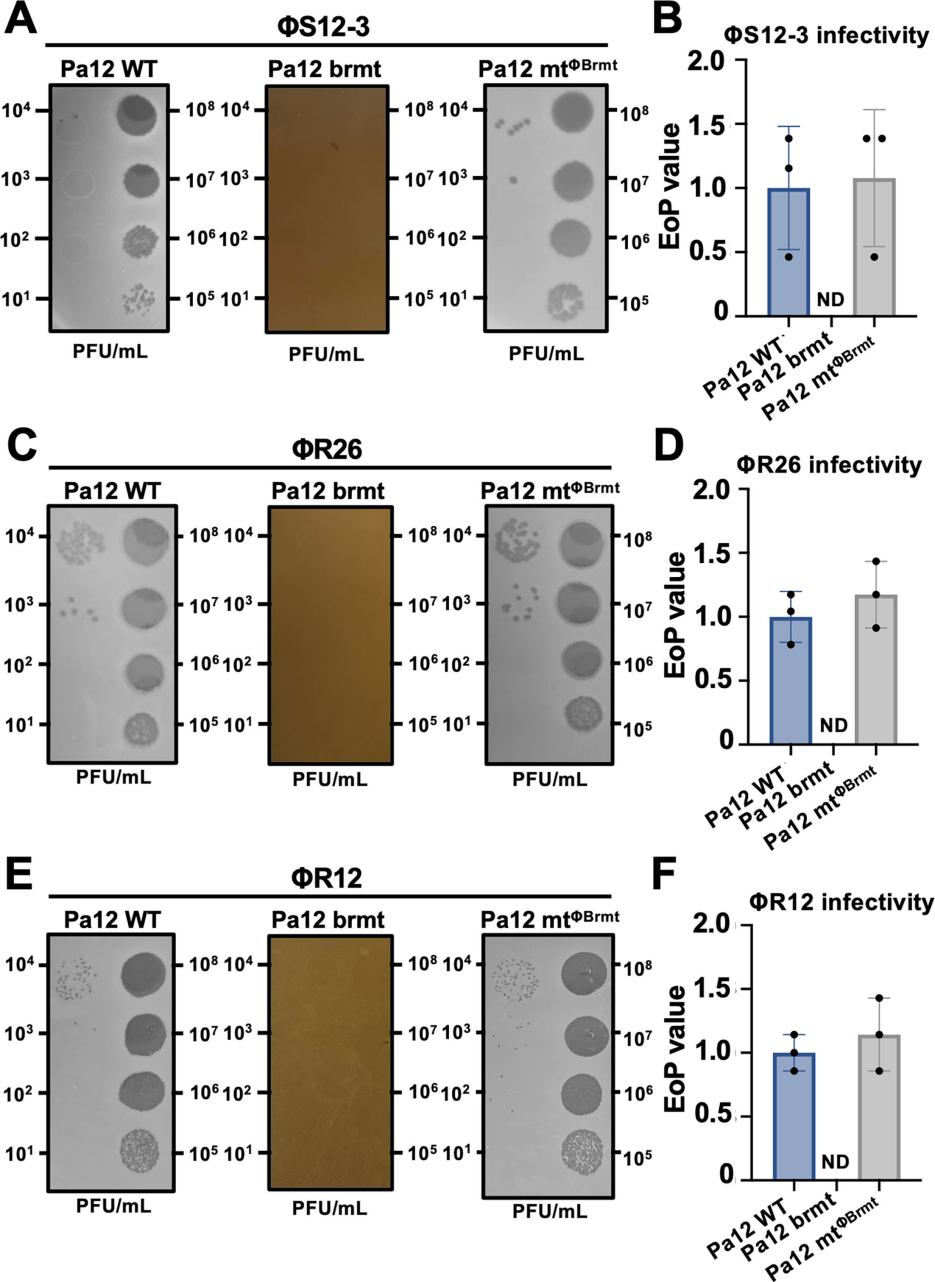
*Pbunavirus* phage infectivity against Pa12 WT, Pa12 Brmt, and Pa12 mt^ΦBrmt^. (**A and B**) Representative images of EoP assays and EoP values of ΦS12-3 on lawns of Pa12 WT, Pa12 Brmt, and Pa12 mt^ΦBrmt^ −2. (**C and D**) Representative images of EoP assays and EoP values of ΦR12 on lawns of Pa12 WT, Pa12 Brmt, and Pa12 mt^ΦBrmt^ −2. (**E and F**) Representative images of EoP assays and EoP values of ΦR26 on lawns of Pa12 WT, Pa12 Brmt, and Pa12 mt^ΦBrmt^ −2. EoP values are indicated as fold change compared to WT and are presented as means ± SD (*n* = 3). ND, plaques not detected.

As shown in Table 1, Pa12 mt^ΦBrmt^ has no mutations in the *O*-antigen associated genes, so we assessed *Pbunavirus* phage infectivity against Pa12 mt^ΦBrmt^. As shown in Fig. 5, EoP assays using *Pbunavirus* ΦS12-3, ΦR12, and ΦR26 revealed that these *Pbunavirus* phages never infect Pa12 Brmt strains; however, Pa12 mt^ΦBrmt^ still possesses the same susceptibility to *Pbunavirus* ΦS12-3, ΦR26, and ΦR12 as Pa12 WT (Fig. 5A through F). These results indicate that the receptor-specific infectivity profiles observed in PAO1 knockout mutants (Fig. 4) are similarly reproduced in mutant strains harboring analogous receptor gene mutations.

### Effects of phage cocktails composed of flagella/pili-targeting ΦBrmt and LPS-targeting *Pbunavirus* phages on *P. aeruginosa* phage resistance

**Fig 6:**
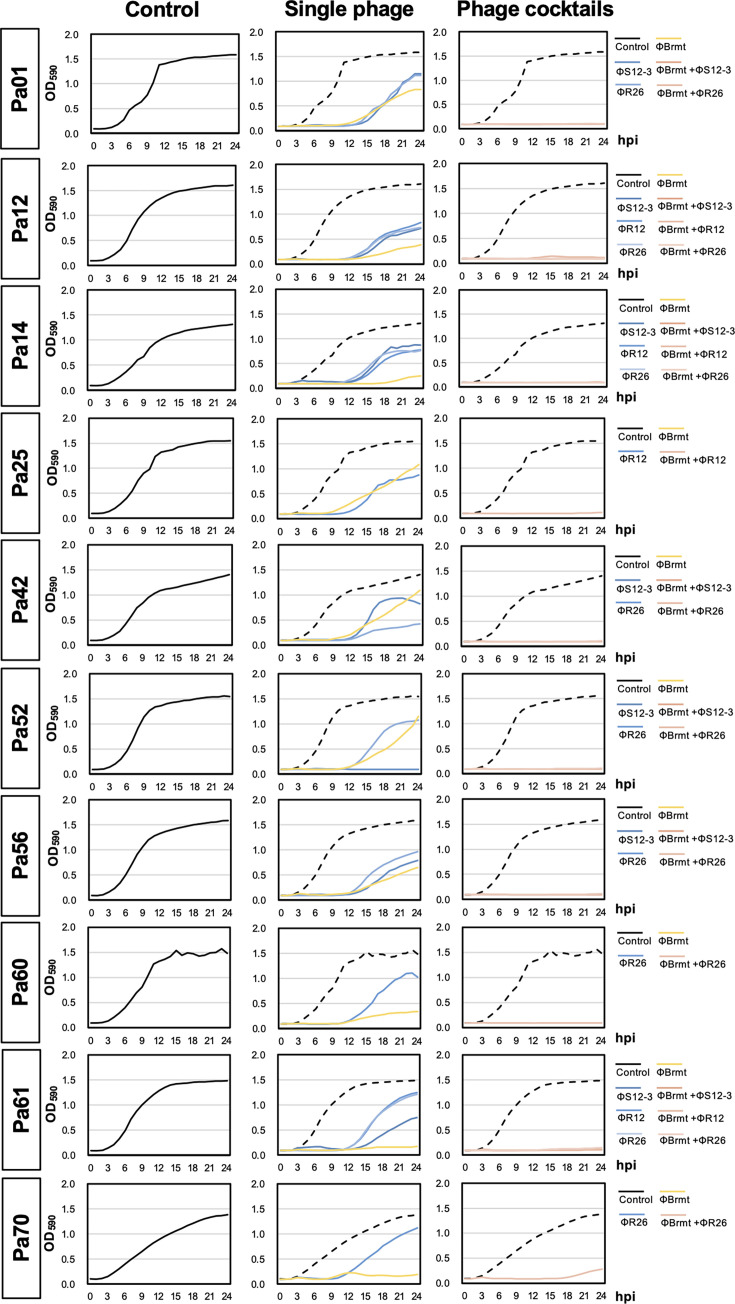
Monitoring the growth of *P. aeruginosa* veterinary isolates with phage cocktails for 24 h. The bactericidal activity of *Pbunavirus* phages (ΦS12-3, ΦR12, and ΦR26), ΦBrmt, and their combinations at MOI of 0.01 were monitored for 24 h. Left panels show growth curves of vehicle controls (SM buffer, *n* = 3). Middle panels show growth curves of single phage inoculations (*n* = 3). Right represent growth curves of phage cocktails composed of *Pbunavirus* phages and ΦBrmt (*n* = 3).

Finally, we examined the effects of phage cocktails on 10 *P*. *aeruginosa* veterinary isolates that were susceptible to ΦBrmt and *Pbunavirus* phages (ΦS12-3, ΦR12, and ΦR26) as shown in Table 2. The growth of *P. aeruginosa* strains in the presence of phage cocktails or single phages was monitored for 24 hpi using a plate reader. As shown in Fig. 6, single phage inoculations (ΦBrmt, ΦS12-3, ΦR12, and ΦR26 only) did not suppress growth curves and allowed *P. aeruginosa* strains to increase their OD value at around 12 hpi, except for Pa52, Pa61, and Pa70, among which ΦBrmt or ΦS12-3 continuously suppressed bacterial growth. In contrast, phage cocktails clearly delayed growth of target strains at 24 hpi, including for Pa01, Pa12, Pa14, Pa25, Pa42, Pa56, Pa60, and Pa61. In the case of Pa70, phage cocktails clearly showed a suppressive effect on phage resistance within 19 hpi, and after that the OD_590_ increased very slightly. Furthermore, we evaluated the efficacy of the phage cocktail up to 48 hpi (Fig. S2A). While an increase in OD_590_ values was observed in strains Pa01 and Pa25 at 48 hpi compared to 24 hpi, the OD_590_ values remained significantly lower than those of the phage-free control group. In contrast, for strains such as Pa12, Pa42, Pa52, Pa56, Pa60, Pa61, and Pa70, certain phage combinations permitted slight bacterial growth after 24 hpi; however, other combinations maintained OD_590_ values below 0.25 even at 48 hpi, indicating sustained suppression of bacterial proliferation. Evaluation of viable *P. aeruginosa* cells at these endpoints revealed that, in all cocktail conditions, bacterial counts were markedly lower than those of the control (Fig. S2B). These results suggest that phage cocktails composed of phages recognizing distinct receptor classes can effectively suppress the growth of target *P. aeruginosa* strains and delay the emergence of phage-resistant variants.

## DISCUSSION

A central challenge in clinical phage therapy is the rapid emergence of phage resistance (6, 9, 10), which underscores the necessity for the rational design of phage cocktails that can create an evolutionary dilemma for the target pathogen. The key of this strategy is to combine phages that target distinct classes of bacterial receptors (9, 10), thereby forcing the bacteria into a state where resistance to one phage may not confer resistance to the other, as described in Fig. 5. For *P. aeruginosa*, while the potent LPS-targeting *Pbunavirus* phages are valuable clinical candidates (11, 13), an over-reliance on them creates a bottleneck. To overcome this, the isolation of phages targeting alternative, non-LPS receptors is a key component. To address this, we performed a strategic screening platform utilizing the LPS-defective *P. aeruginosa* Brmt mutant (17, 18), as similar to a previous report, which has shown that isolation of novel phages using phage-resistant variants (36). This approach successfully led to the isolation of ΦBrmt. In fact, the Brmt strain’s distinct brown phenotype serves as a powerful visual marker, enabling a rapid, culture-based pre-screening for non-LPS-targeting phages. This methodology circumvents the need for laborious, time-consuming genomic analyses at the initial isolation stage. Ultimately, our work not only provides a novel phage candidate (ΦBrmt) that is an ideal orthogonal partner for LPS-targeting phages but also establishes a streamlined and accelerated isolation pipeline for identifying phages with diverse receptor specificities, paving the way for more robust and resistance-proof cocktail therapies against *P. aeruginosa*.

The flagellar structure of *P. aeruginosa* has long been implicated as a phage receptor. For instance, phage BTX was considered the first flagella-targeting phage, as its infectivity was significantly reduced by anti-flagellin antibodies and flagellar peptides (24). Furthermore, recent transposon library screenings have correlated the loss of flagellar gene function with phage resistance, suggesting that flagella serve as a major receptor for a certain range of *P. aeruginosa* phages (22). However, our findings for ΦBrmt suggest a significant departure from these reports. While our transmission electron microscopy analysis clearly showed ΦBrmt adsorbing to flagellar filaments, the phage retained its ability to infect a *fliC*, which is encoding filaments, single-knockout mutant. This paradox strongly indicated the involvement of an alternative receptor. Indeed, mutational analysis of ΦBrmt-resistant variants revealed mutations in genes encoding type IV pili—a well-known receptor for jumbo phages (Table 1) (19–21). Crucially, however, neither a *fliC* nor a *pilA* single knockout abolished infectivity; only the Δ*pilA*/Δ*fliC* double knockout rendered the host completely resistant (Fig. 4C). These results suggest that ΦBrmt possesses a novel dual-receptor mechanism, capable of using either pili or flagella as a primary receptor to initiate a successful infection. This unique potential dual-receptor capability may explain the distinct lysis dynamics observed in our experiments (Fig. 6). For two clinical isolates (Pa61 and Pa70), ΦBrmt single-inoculation was significantly more effective at suppressing the emergence of resistance over 24 h than the LPS-targeting phages were. We hypothesize that ΦBrmt, by targeting two distinct motility structures, effectively may act as a “cocktail-in-one,” delaying resistance evolution because the bacterium must simultaneously acquire mutations in two functionally separate systems to escape infection. Indeed, a similar dual-receptor mechanism has been reported for the *Escherichia* phage Bp7, which can use either of the distinct outer membrane proteins LamB or OmpC for infection. Since infection proceeds as long as one receptor is available, it has been suggested that this multi-receptor targeting strategy can delay the emergence of resistant mutants (37). Consequently, the combination of ΦBrmt with an LPS-targeting *Pbunavirus* phage may create an exceptionally potent cocktail. This formulation engages three functionally and structurally distinct sites—LPS, flagella, and pili—presenting a formidable evolutionary barrier that explains the robust suppression of resistance observed in our study. On the other hand, it is important to note that the present study was limited to the suppression of phage resistance *in vitro*. The synergistic efficacy and safety of this phage cocktail must be further validated in more practical settings, including preclinical and clinical trials, to confirm its therapeutic potential.

Phage cocktails are a great option to inhibit phage-resistant variants, and there have been several clinical trials testing pharmaceutically developed phage cocktails (5, 10). In addition to our results (Fig. 6; Fig. S2), many other studies have found that phage cocktails strongly suppress the occurrence of phage-resistant variants compared with single inoculations of phages *in vitro* (36, 38); however, it is possible that phage resistant variants will occur during actual phage therapy as previously reported (6, 9, 10). On the other hand, we can apply desirable selective pressure by using phage therapy, because genetic mutations that cause phage resistance also produce bacterial phenotypical alterations such as decreased pathogenicity and the restoration of antibiotic sensitivity (9, 39, 40). As shown in Table 1 and Fig. 2E, Pa12 mt^ΦBrmt^ lacks a flagellar structure and has significantly decreased swimming and twitching activity (Fig. 3), which reflects pili and flagellar function. As *Pseudomonas* pili play an important role in attachment during infections (41, 42), decreased pilus activity might suppress the adhesion of the bacteria. In addition, the swimming motility of Pa12 mt^ΦBrmt^-2 was significantly decreased compared to Pa12 mt^ΦBrmt^-1 and 3, suggesting that specific genetic mutations in the type IV pilus genes are associated with the motility of phage-resistant variants. Notably, as PilJ is required for complete type IV pilus assembly and extension (43), Pa12 mt^ΦBrmt^-2, which harbors a *pilJ* mutation, exhibited strong motility reduction compared to Pa12 mt^ΦBrmt^-1 and 3, which possess *pilY1* and *pilA* mutations involved in pilus tip formation and pilus filament formation, respectively (44, 45). In addition, *Pseudomonas* flagella are essential to the virulence of the pathogen and are associated with clearance from infection sites (41). It has been reported that *P. aeruginosa flC* mutants cause pneumonia in 25% of infected mice, but parental strains cause it in 80% of mice, suggesting that flagellar dysfunction facilitates clearance (46). Therefore, even if ΦBrmt-resistant variants occur during the phage therapy, these variants might remarkably impair pilus and flagellum function, leading to a decrease in virulence and facilitating clearance. Indeed, the clinical utility of targeting such motility structures is underscored by the remarkable success of recent trials using pili-targeting *Pseudomonas* phages (47). This highlights that a forward-looking approach to cocktail design—one that strategically considers the evolutionary trade-offs of resistance—will be a key determinant of future therapeutic success.

In conclusion, we characterized *Phikzvirus* ΦBrmt, which was isolated by using the *O*-antigen-defective *P. aeruginosa* strain Pa12 Brmt. Genetic and electron-microscopic evidence strongly indicated that ΦBrmt infects *P. aeruginosa* in an *O*-antigen-independent manner and utilizes flagella and pili structure as a primary receptor. A phage combination of ΦBrmt with LPS-targeting phages such as *Pbunavirus* ΦS12-3, ΦR12, and ΦR26 clearly suppressed the occurrence of phage-resistant variants compared with single phage inoculation, suggesting that these phage cocktails are promising candidates for phage therapy. Therefore, identifying the receptor genes utilized by *Pseudomonas* phages can be the rational starting point for such design. In addition, even if phage-resistant variants occur during therapy, ΦBrmt resistance might result in *P. aeruginosa* phenotypes with decreased virulence and faster clearance. Our findings expand our understanding of phage cocktail construction and phage therapy for *Pseudomonas* infection.

## Data Availability

The data sets generated during and/or analyzed during the current study are available from the corresponding author on reasonable request. DNA sequences of phages (ΦBrmt, ΦR12, ΦS12-3, ΦR26) are available in the DDBJ/EMBL/GenBank databases under accession numbers LC727695.1, NC_048662.1, LC472883.1, and NC_048663.1. In addition, the DNA sequence of the host strain (Pa12) is available in the DDBJ/EMBL/GenBank databases under accession number NZ_AP024513.1.

## SUPPLEMENTAL MATERIAL

Supplemental figures aem.02095-25-s0001.pdf Figures S1 and S2.

Supplemental tables aem.02095-25-s0002.xlsx Tables S1 to S3.

ASM does not own the copyrights to Supplemental Material that may be linked to, or accessed through, an article. The authors have granted ASM a non-exclusive, world-wide license to publish the Supplemental Material files. Please contact the corresponding author directly for reuse.
