# Supplementary material for: Phage cocktails containing a dual-receptor Phikzvirus suppress resistance evolution in Pseudomonas aeruginosa
Source: Appl Environ Microbiol. 2026 Jan 27;92(2):e02095-25. doi: 10.1128/aem.02095-25 (PMC12915314; doi:10.1128/aem.02095-25)
Supplement: Supplemental figures — Figures S1 and S2. [file aem.02095-25-s0001.pdf]

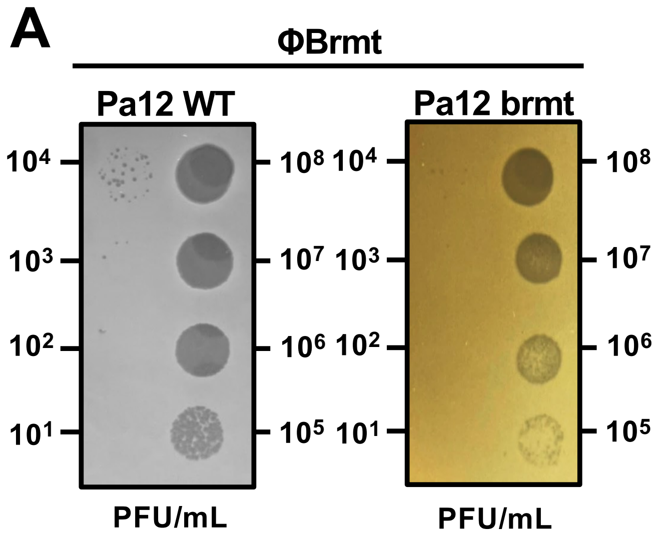

Figure S1. ΦBrmt infectivity against Pa12 WT and Brmt strains.

ΦBrmt infectivity against Pa12 WT and Brmt strains was evaluated by the EoP method. Representative images of EoP assays and plaques produced by ΦBrmt on a lawn of Pa12 WT and Brmt strains.

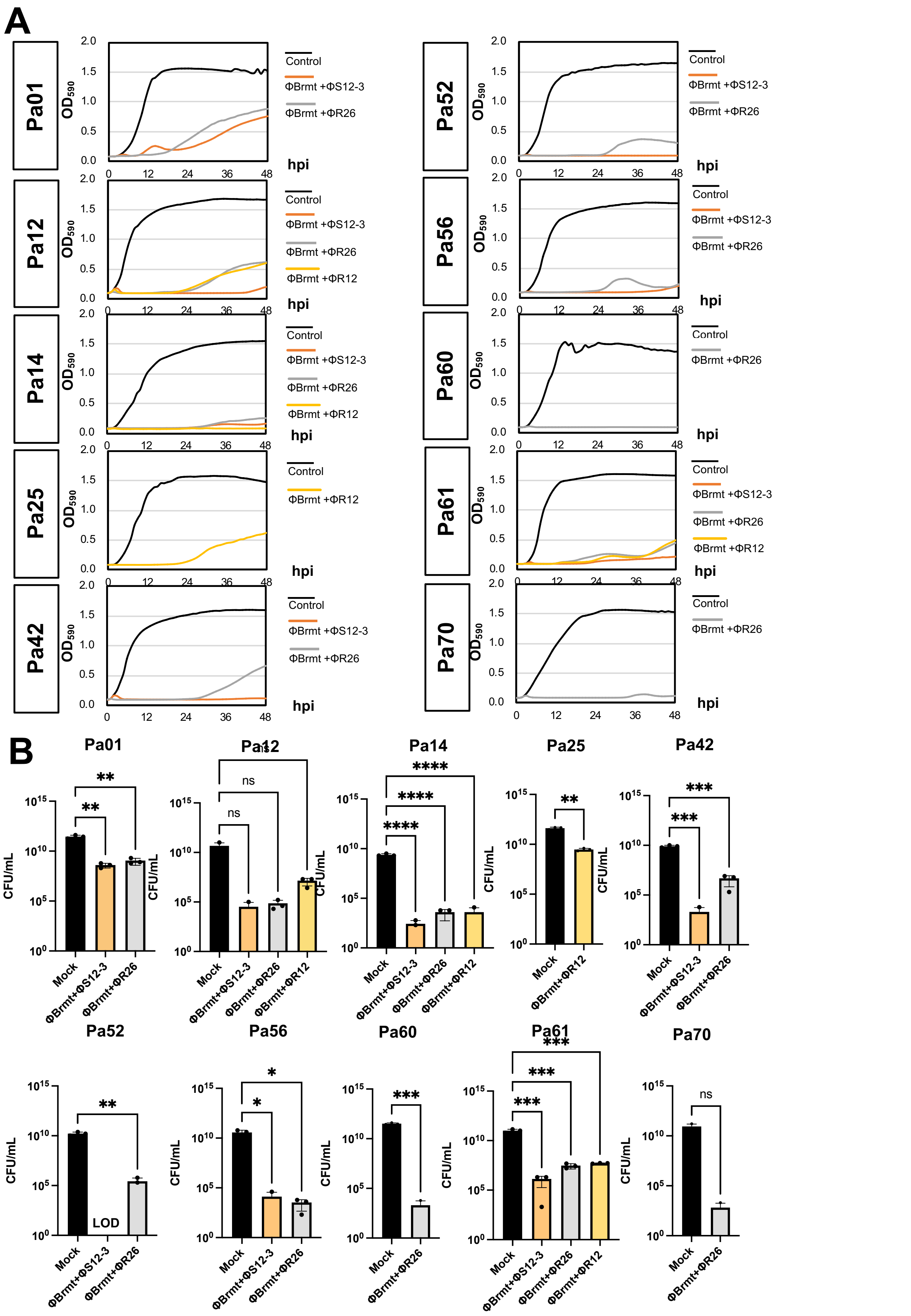

Figure S2. Monitoring the growth of* P. aeruginosa *veterinary isolates with phage cocktails for 48 hours.

(A) Combinations of Pbunaviruses (ΦS12-3, ΦR12, and ΦR26) and ΦBrmt at an MOI of 0.01 were monitored for 48 h (n = 3). (B) Levels of viable P. aeruginosa cells after 48 h of treatment with phage cocktails. Values are presented as mean CFU/mL ± SD (n = 3). Statistical significance compared to the vehicle control (Mock) was determined by Dunnett's test based on a one-way ANOVA: * *p*<0.05, ** *p*<0.01, *** *p*<0.001, and **** *p*<0.0001.
